# Anastomosing Haemangioma of the Kidney Involving a Segmental Branch of the Renal Vein

**DOI:** 10.1155/2015/927286

**Published:** 2015-09-07

**Authors:** Ayodeji Oluwarotimi Omiyale, Anurag Golash, Amandeep Mann, Dimitris Kyriakidis, Karthik Kalyanasundaram

**Affiliations:** ^1^Department of Histopathology, Maidstone and Tunbridge Wells NHS Trust, Maidstone Hospital, Maidstone, Kent ME16 9QQ, UK; ^2^Department of Urology, University Hospital of North Midlands NHS Trust, Royal Stoke University Hospital, Stoke-on-Trent ST4 6QG, UK; ^3^Department of Histopathology, Walsall Healthcare NHS Trust, Manor Hospital, Walsall WS2 9PS, UK; ^4^Department of Histopathology, University Hospital of North Midlands NHS Trust, Royal Stoke University Hospital, Stoke-on-Trent ST4 6QG, UK

## Abstract

Anastomosing variant of capillary haemangioma is a rare and recently described vascular tumour with a proclivity for the genitourinary tract. Here we present the case of a 64-year-old man with incidental finding of 3.4 cm renal mass on CT who had laparoscopic nephrectomy with a good postoperative recovery. Histopathological diagnosis of anastomosing haemangioma of the kidney was made and the patient was followed up for 10 months without evidence of tumour recurrence.

## 1. Introduction

Vascular tumours of the kidney are very rare. Anastomosing haemangioma of the kidney is a subtype of capillary haemangioma. We describe a case of anastomosing haemangioma in a 64-year-old male patient with the CT imaging findings, histopathological features, treatment, and outcome.

## 2. Case Presentation

A 64-year-old male who had suffered a deep vein thrombosis of the left lower limb underwent an ultrasound, which revealed an incidental finding of a 2.5 cm lesion involving the left kidney. His past medical history included type 2 diabetes mellitus and peripheral vascular disease with claudication. He had no haematuria or weight loss.

**Figure 1:**
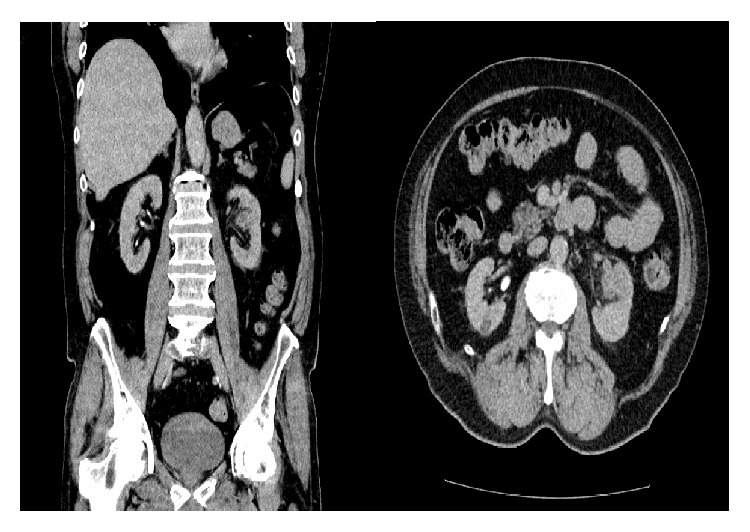
CT imaging of the mid pole solid mass of the left kidney.

A CT abdomen-pelvis with contrast confirmed a suspicious 3.4 cm left mid pole enhancing renal mass with involvement of the segmental renal vein (Figure 1). There was apparent involvement of the anterior calyx of the left mid pole complex. There was no significant renal hilar, paracaval, and para-aortic adenopathy. The mass was suggestive of renal cell carcinoma. A completion CT of the chest did not show any lesion or metastasis.

The results were discussed in the multidisciplinary team meeting and consequently with the patient and he agreed to proceed to a laparoscopic nephrectomy, which was performed a week later.

**Figure 2:**
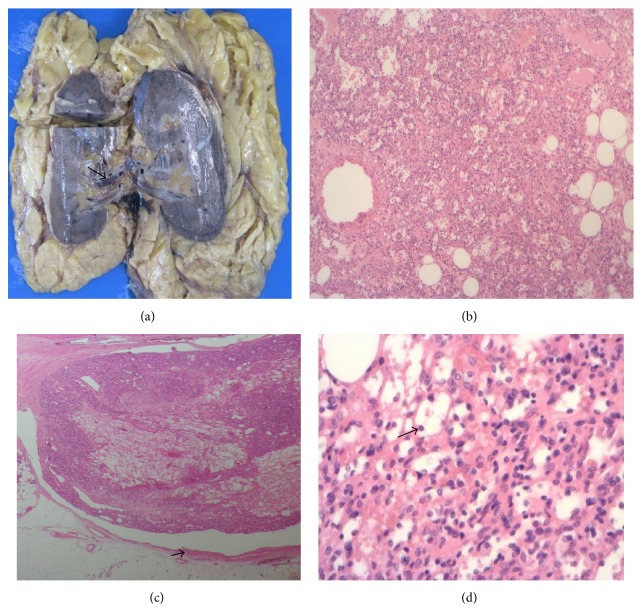
(a) Macroscopic image of the tumour in the renal hilum with involvement of the branch of a segmental renal vein (arrowed). (b) 40x H&E showing the tumour with sieve-like pattern. (c) 20x H&E demonstrating the involvement of the tributary of a segmental renal vein (vessel wall arrowed). (d) 200x H&E showing round to oval vasoformative endothelial cells with hobnail appearance (arrowed).

**Figure 3:**
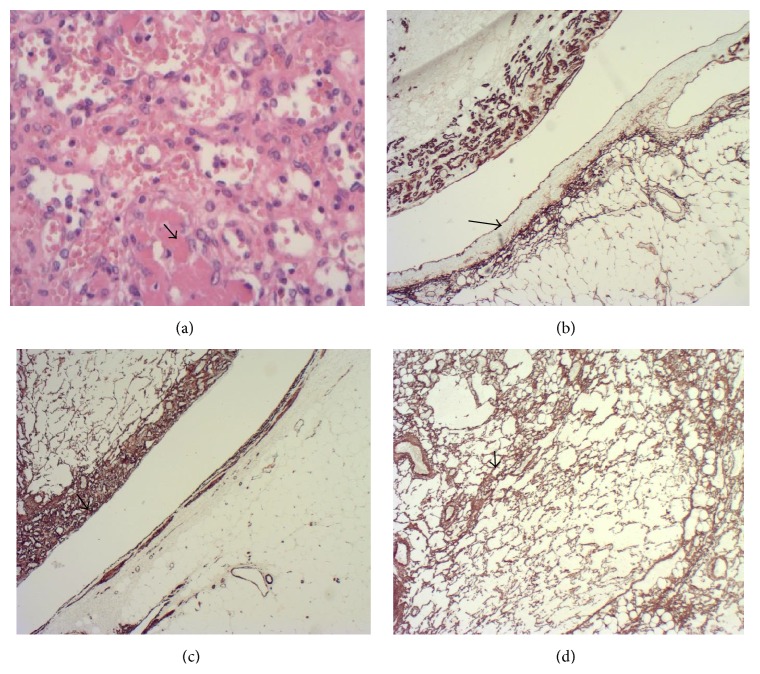
(a) 200x H&E showing fibrin thrombi within some vascular spaces. (b) showing CD34 immunohistochemistry staining the endothelium in the tumour as well as the segmental renal vein branch (arrowed) (×20). (c) demonstrating smooth muscle actin immunohistochemistry staining the tumour pericytes (arrowed) and vessel wall (×20). (d) showing CD34 immunohistochemistry staining supporting pericytes (×40).

Grossly, the renal lesion measured 2.3 × 2.4 × 1.8 mm was lobulated and mahogany-brown in colour. Further macroscopic assessment showed a slightly haemorrhagic tumour located in the renal hilum and renal sinus fat, with a portion of the tumour identified within the branch of a renal segmental vein (Figure 2(a)). Microscopic examination showed a lobulated vasoformative tumour which had a “sieve-like” anastomosing vascular pattern composed of small to medium sized vessels (Figure 2(b)). Extension into the tributary of a segmental renal vein was confirmed on microscopy (Figure 2(c)). Higher magnification showed thin-walled vascular channels lined by round to oval endothelial cells with hobnail appearance (Figure 2(d)). Intravascular fibrin thrombi were identified in some vascular spaces (Figure 3(a)). There was no evidence of coagulative tumour necrosis and renal parenchymal invasion. No cytological atypia was identified.

On immunohistochemistry, the tumour was positive with CD34 and CD31. The endothelium of the renal vein branch demonstrated CD34 positivity (Figure 3(b)) and the muscular wall of the vein was positive for smooth muscle actin (Figure 3(c)). Smooth muscle actin also delineated the supporting pericytes within the tumour (Figure 3(d)). There was negative staining with Cam 5.2, MNF116, EMA, HMB45, CD117, synaptophysin, chromogranin, CD10, RCC, 34BE12, and S-100. MIB-1 showed low proliferative index. The overall appearance was suggestive of anastomosing haemangioma, but a second opinion from an external laboratory was requested, which also confirmed the diagnosis. This patient recovered very well postoperatively and has been followed up for 10 months without signs of tumour recurrence.

## 3. Discussion

Anastomosing haemangioma is a morphological variant of capillary haemangioma described by Montgomery and Epstein. The term “anastomosing haemangioma” describes the histological observation of anastomosing pattern of capillary sized vascular channels reminiscent of splenic sinusoids [1].

The incidence of the tumour in male patients suggests a slight male predilection with a reported male to female ratio of 1.8 : 1 [2]. The average age of presentation is 56 years and the lesions are mostly unilateral. The clinical presentation is nonspecific, which includes haematuria, abdominal pain, and lower urinary tract symptoms (LUTs). The tumour may be an incidental finding during the workup for unrelated clinical conditions [3] as observed in our patient.

Grossly, anastomosing haemangioma of the kidney is often small sized with an average size of 1.8 cm [4]. The tumour is haemorrhagic or mahogany-brown in colour, with well-demarcated margins and a spongy consistency. Anastomosing haemangioma lacks features of malignancy such as increased mitotic activity, nuclear atypia, multilayering, and endothelial tufting [4–6].

The tumour typically stains positive with endothelial markers CD31 and CD34 and factor VIII-related protein [1, 4, 5]. Contrast-enhanced CT imaging cannot reliably differentiate anastomosing haemangioma from other aggressive renal neoplasms such as angiosarcoma and renal cell carcinoma; consequently anastomosing haemangioma is often underdiagnosed prior to surgery and usually treated with nephrectomy [5, 6]. The tumour runs a benign course without evidence of tumour recurrence reported in the literature.

## 4. Conclusion

We have presented a case of anastomosing haemangioma of the kidney which is a recently described vascular tumour which runs a benign course. This case is of particular note because of the gross tumour involvement of a segmental branch of the left renal vein which was identified on CT imaging and confirmed microscopically. This case expands the knowledge of this rare tumour and emphasizes the importance of histopathological diagnosis in ruling out more aggressive differential diagnosis which has implications on further management.
